# Metabolic regulation of T cell production of IL-10 and IL-22 protects against intestinal inflammation

**DOI:** 10.1093/pcmedi/pbaf025

**Published:** 2025-10-24

**Authors:** Han Liu, Xiaojing Zhao, Tianming Yu, Yu Yu, Suxia Yao, Wenjing Yang, Yingzi Cong

**Affiliations:** Division of Gastroenterology and Hepatology, Department of Medicine, Northwestern University, Chicago, IL 60611, USA; Center for Human Immunobiology, Northwestern University, Chicago, IL 60611, USA; Department of Microbiology and Immunology, University of Texas Medical Branch, Galveston, TX 77555, USA; Division of Gastroenterology and Hepatology, Department of Medicine, Northwestern University, Chicago, IL 60611, USA; Center for Human Immunobiology, Northwestern University, Chicago, IL 60611, USA; Department of Microbiology and Immunology, University of Texas Medical Branch, Galveston, TX 77555, USA; Department of Microbiology and Immunology, University of Texas Medical Branch, Galveston, TX 77555, USA; Division of Gastroenterology and Hepatology, Department of Medicine, Northwestern University, Chicago, IL 60611, USA; Center for Human Immunobiology, Northwestern University, Chicago, IL 60611, USA; Department of Microbiology and Immunology, University of Texas Medical Branch, Galveston, TX 77555, USA; Division of Gastroenterology and Hepatology, Department of Medicine, Northwestern University, Chicago, IL 60611, USA; Center for Human Immunobiology, Northwestern University, Chicago, IL 60611, USA; Department of Microbiology and Immunology, University of Texas Medical Branch, Galveston, TX 77555, USA; Division of Gastroenterology and Hepatology, Department of Medicine, Northwestern University, Chicago, IL 60611, USA; Center for Human Immunobiology, Northwestern University, Chicago, IL 60611, USA; Department of Microbiology and Immunology, University of Texas Medical Branch, Galveston, TX 77555, USA; Department of Microbiology-Immunology, Northwestern University, Chicago, IL 60611, USA; Department of Pathology, Northwestern University, Chicago, IL 60611, USA

**Keywords:** T cell metabolism, IL-10, IL-22, dichloroacetate, Ahr, Bhlhe40

## Abstract

**Objectives:**

Inflammatory bowel disease is driven by dysregulated CD4⁺ T cell responses to the intestinal microbiota. While T cells can exacerbate inflammation by producing proinflammatory cytokines, they also produce anti-inflammatory mediators, such as interleukin 10 (IL-10) and IL-22. However, the metabolic programs that regulate IL-10 and IL-22 production remain incompletely defined.

**Methods:**

We used CBir1 transgenic mice and *in vitro* Th1 polarization assays to investigate how metabolic pathways regulate T cell production of IL-10 and IL-22. A panel of metabolic inhibitors was tested for their effects on cytokine expression. Transcriptional mechanisms were assessed using bulk RNA sequencing, qPCR, Enzyme-linked immunosorbent (ELISA), and CRISPR-Cas9–mediated gene editing. Functional relevance was validated using *Citrobacter rodentium* infection and T cell suppression assays *in vivo* and *in vitro*.

**Results:**

Among tested metabolic inhibitors, dichloroacetate (DCA) significantly enhanced IL-10 and IL-22 production by CD4⁺ T cells. DCA increased maximal oxygen consumption and decreased lactate secretion in T cells. Mechanistically, DCA upregulated aryl hydrocarbon receptor (*Ahr*) and downregulated *Bhlhe40*, without affecting *Prdm1*. Pharmacologic inhibition of Ahr suppressed DCA-induced IL-22, but not IL-10, while Bhlhe40 knockout enhanced IL-10 production, identifying distinct transcriptional regulators for each cytokine. Functionally, DCA-treated Th1 cells suppressed naïve T cell proliferation via IL-10. In an *in vivo* experiment, DCA treatment protected mice from *C. rodentium-*induced colitis.

**Conclusions:**

Our findings demonstrate that DCA enhances IL-22 and IL-10 production in Th1 cells through Ahr and Bhlhe40, respectively. These results identify a novel metabolic mechanism by which DCA promotes mucosal immune regulation and highlight its potential as a therapeutic strategy for inflammatory bowel disease.

## Graphical Abstract

**Graphical Abstract:**
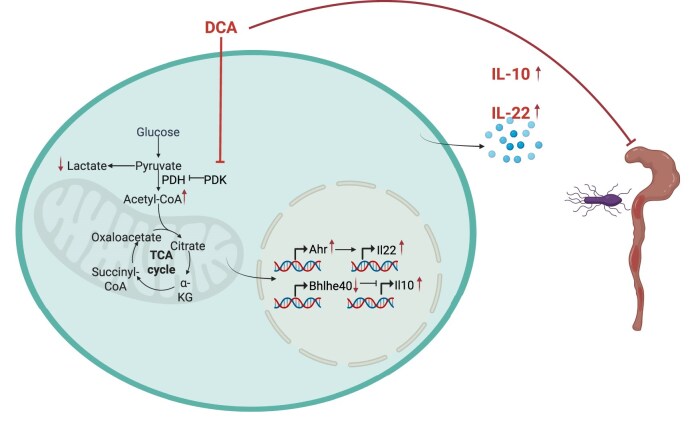
Dichloroacetate (DCA) promotes the conversion of pyruvate to acetyl-CoA, enhancing mitochondrial metabolism. This shift upregulates IL-10 and IL-22 via Bhlhe40 and AhR, respectively. *In vivo*, DCA suppresses Citrobacter rodentium-induced colitis.

## Introduction

Inflammatory bowel disease (IBD), which consists of ulcerative colitis (UC) and Crohn’s disease (CD), represents a chronic and recurrent inflammatory disorder that primarily affects the intestines [1]. It has been well-established that the pathogenesis of IBD is mediated by an overactive response of CD4^+^ T cells to the intestinal microbiota, resulting in impaired mucosal immunity [2–4]. Abnormal Th1 and Th17 cell responses to gut microbiota, which have been shown to contribute to the pathogenesis of IBD [5–7], are controlled by several mechanisms, including T cell-producing interleukin 10 (IL-10) and IL-22. IL-10 is one of the anti-inflammatory cytokines produced by T regulatory (Treg) cells that contributes to intestinal homeostasis. Additionally, IL-10 produced by T effector cells is also considered a suppressive factor in restraining intestinal inflammation. IL-22 was originally identified as a Th1 cytokine, but was later found to be produced by Th17 and Th2 as well [8–11]. IL-22 is crucial to host protective responses against inflammatory insults at the intestinal epithelial barrier. IL-22 can also be produced by innate lymphoid cells (ILCs). Several recent reports indicate that whereas ILCs act as an early source of IL-22 that provides rapid protection to the intestinal epithelial barrier, T cells emerge as the predominant source of IL-22 during chronic inflammation [12]. However, the mechanisms by which T cells produce IL-10 and IL-22 are still not completely understood.

It is now well-established that each T cell subset utilizes a metabolic program specific to its function. Th17 and Th1 cells primarily rely on upregulated aerobic glycolysis to meet their metabolic requirements, and Treg cells rely predominantly on mitochondrial oxidation for energy and biosynthetic precursors [13–17]. However, the specific metabolic programs that regulate T cell production of IL-10 and IL-22 have yet to be defined.

The mitochondrial pyruvate dehydrogenase (PDH) complex converts pyruvate into acetyl-CoA (Ac-CoA), which enters the tricarboxylic acid cycle to produce energy ATPs in the OXPHOS pathway [18, 19]. PDH complex activity is negatively regulated by pyruvate dehydrogenase kinases (PDHK) [20, 21], a family of four isoforms (PDHK1–4) with variable expression reported in various cells [22]. PDHK promotes aerobic glycolysis by phosphorylating and inactivating PDH. Dichloroacetate (DCA), a small molecule that inhibits PDHK 1–4 activity [23], has been investigated as a metabolic-targeting therapy for various conditions, including diabetes, glioblastoma multiforme, and genetic mitochondrial disorders [24–26]. Recent research has highlighted its therapeutic potential in a mouse model of UC, where it modulates the NFATC1/NLRP3/IL1B signaling pathway to alleviate inflammation [27]. DCA promotes the oxidation of pyruvate by enhancing its entry into the tricarboxylic acid cycle, thereby limiting its conversion into lactate [28]. DCA decreases T cell metabolism with a greater effect on glycolysis than on OXPHOS [29]. DCA has recently been reported to inhibit Th1 and Th17 cell development and inflammatory diseases [30]. However, it is still unclear if DCA regulates T cell production of IL-10 and IL-22 to inhibit intestinal inflammation.

This study reveals that DCA significantly enhances IL-10 and IL-22 secretion in Th1 cells, highlighting its potent immunoregulatory effects. DCA treatment effectively suppressed the intestinal inflammation induced by *Citrobacter rodentium* infection. Furthermore, we identify aryl hydrocarbon receptor (Ahr) as a key mediator of DCA-induced IL-22 production, while Bhlhe40 mediates DCA-induced IL-10 production. These findings provide novel metabolic mechanisms involved in T cell production of IL-10 and IL-22, paving the way for DCA as a promising therapeutic application in IBD.

## Methods

### Animals

C57BL/6J mice were purchased from The Jackson Laboratory and maintained in a specific pathogen-free facility under controlled conditions (20–26 °C, 30%–70% humidity, 12-h light/dark cycle). Animal experiments were approved by the respective Institutional Animal Care and Use Committee (IACUCs) at the University of Texas Medical Branch (approval No. 2 004 044) and Northwestern University (approval No. IS00027174) and performed in accordance with institutional animal care policies.

### Isolation and culture of CD4^+^ T cells

CD4⁺ T cells were purified from spleens using anti-mouse CD4 magnetic microbeads and cultured in 24-well plates either with anti-CD3 (5 µg/ml) or anti-CD28 (2 µg/ml) antibodies. Alternatively, cells were activated with splenic antigen-presenting cells (APCs) and CBir1 peptide (Thermo Fisher Scientific). Th1 cells were polarized by adding recombinant IL-12 (10 ng/ml). To dissect metabolic regulation, cultures were treated with various metabolic inhibitors, including DCA (1–50  mM), 2-deoxy-D-glucose (2-DG, 0.5 Mm), etomoxir (50 µM), 6-diazo-5-oxo-L-norleucine (DON, 1 µM), metformin (10 µM), oligomycin (0.5 µM), or benzodiazepine-423 (Bz-423) (15 µM).

### RNA sequencing

CD4⁺ T cells cultured under Th1 conditions with or without 10 mM DCA for 48 h were harvested for RNA extraction. RNA quality was verified by Bioanalyzer (Agilent), and high-integrity samples were submitted to Novogene for library construction and sequencing. mRNA was enriched via oligo(dT) selection, randomly fragmented, and reverse-transcribed into first-strand cDNA using random hexamer primers. The second strand was synthesized with DNA polymerase I and RNase H. After end repair, poly-A tailing, adaptor ligation, and PCR enrichment, libraries with 250–350 bp inserts were sequenced on an Illumina NovaSeq 6000 platform (paired-end, 150 bp reads). Raw sequencing reads were processed using the Novosmart pipeline for quality control, alignment to the mouse reference genome (mm10), and differential gene expression analysis.

### Quantitative real-time PCR

Total RNA was isolated from CD4⁺ T cells with TRIzol, followed by reverse-transcription using a qScript cDNA Synthesis Kit. Gene levels were detected using SYBR Green-based real-time PCR on a CFX96 Touch system. Primer pairs are listed in supplementary Table 1 (see online supplementary material).

### Enzyme-linked immunosorbent assay

CD4⁺ T cell culture supernatants were collected at day 3, and IL-10 and IL-22 were measured using Enzyme-linked immunosorbent (ELISA). The plates (Thermo Fisher) were coated at 4°C overnight with capture antibodies for mouse IL-10 (Clone JES5-2A5, BioLegend, Cat#505 010) or IL-22 (Clone Poly5164, Invitrogen, Cat#14–7221-82). After blocking with 1% bovine serum albumin in phosphate-buffered saline, the samples and standards were added for incubation for 2 h at room temperature. Biotinylated detection antibodies and streptavidin-Horseradish peroxidase were used for signal amplification, followed by 3,3′,5,5′-tetramethylbenzidine substrate (BD Biosciences, Cat#555 214). Optical density at 450 nm was measured using a BioTek Synergy plate reader.

### Flow cytometry

Cells were treated with phorbol 12-myristate 13-acetate (50 ng/ml) and ionomycin (750 ng/ml) for 2 h, followed by treatment with brefeldin A (1 µl/ml) for 3 h. Cells were stained with live/dead stain and surface-stained for CD4. Intracellular cytokines were detected after fixation/permeabilization using antibodies against IL-10, IL-22, interferon (IFN)-γ, and IL-17A. Cells were acquired using BD Symphony SE, and the was data analyzed with FlowJo software.

### Mitochondrial oxygen consumption

T cells (200K/well) were seeded onto 96-well plates and oxygen consumption was determined using a Mito Stress kit by Seahorse Analyzer.

### *Citrobacter rodentium* infection

Mice were gavaged with *C. rodentium* (5 × 10⁸ Colony-forming unit (CFU)/mouse; strain DBS100, ATCC) on day 0. DCA (5 mg/mouse) was administered via oral gavage. On day 7, feces were collected and cultured on MacConkey agar (BD Biosciences) for bacterial enumeration.

Colonic and cecal tissues were harvested from mice 10 days after *C. rodentium* infection and prepared as Swiss rolls. Samples were fixed in 10% neutral-buffered formalin for 24 h, embedded in paraffin, and sectioned at 5 µm thickness. Sections were stained with hematoxylin and eosin (H&E) for histopathological evaluation. To objectively quantify intestinal inflammation, a composite scoring system was employed that reflects multiple aspects of mucosal pathology.

### Statistical analysis

Statistical analyses were performed using GraphPad Prism version 10.0. Data are presented as mean ± standard error of the mean (SEM). Unpaired two-tailed Student’s t-test or Mann–Whitney U test was performed for statistical comparisons between the two groups. For multiple group comparisons, one-way analysis of variance (ANOVA) with Bartlett’s or Tukey’s *post hoc* test was used. Statistical significance was defined as *P* < 0.05.

## Results

### Metabolic regulation of T cell production of IL-10 and IL-22

**Figure 1.**
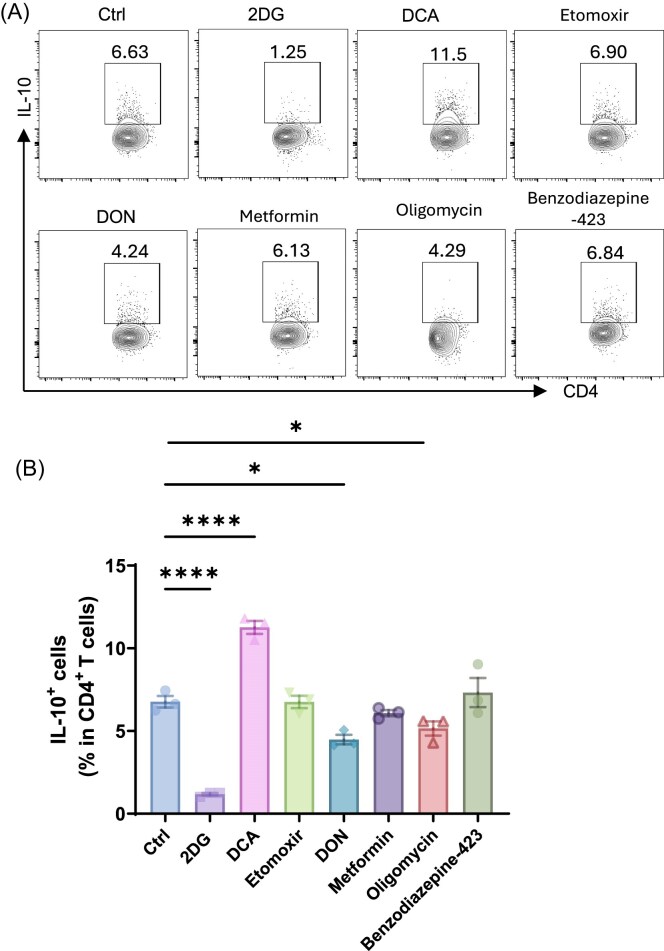
DCA enhances IL-10 production in Th1 cells by metabolic reprogramming. Splenic CD4⁺ T cells from CBir1 T cell receptor (TCR) transgenic mice were cultured under Th1-polarizing conditions with various metabolic inhibitors (Ctrl: control; 0.5 mM 2DG; 1 mM DCA; 50 µm etomoxir; 1 µm DON; 10 µm metformin; 0.5 µm oligomycin; 15 µm Bz-423) for 5 days (*n* = 3/group). (**A**) Intracellular IL-10 expression was analyzed by flow cytometry. (**B**) Frequency of IL-10⁺ CD4⁺ T cells was quantified from three independent experiments. Representative of three experiments. Data are presented as mean ± SEM. One-way ANOVA with Bartlett’s multiple comparisons test (B). *P < 0.05, ^****^P < 0.0001.

To investigate how metabolic pathways regulate IL-10 production in Th1 cells, CD4⁺ T cells from spleens of CBir1 transgenic (tg) mice, specific for gut microbiota antigen CBir1 flagellin [31], were stimulated with irradiated APCs pulsed with CBir1 peptide under Th1-polarizing conditions in the presence of various metabolic inhibitors, including 2DG (a glycolysis inhibitor), DCA (a pyruvate dehydrogenase kinase inhibitor), etomoxir (a fatty acid oxidation inhibitor), DON (a glutaminolysis inhibitor), metformin (a mitochondrial complex I inhibitor), oligomycin (an ATP synthase inhibitor), and Bz-423 (a mitochondrial modulator targeting ATP synthase). After 5 days, IL-10 expression was analyzed by flow cytometry (Fig. 1).

Among these metabolic inhibitors, 2DG and DON markedly suppressed IL-10 expression (Fig. 1B). Interestingly, both Bz-423 and oligomycin target mitochondrial ATP synthase, but the effect on IL-10 production differs. Oligomycin suppressed IL-10, but Bz-423 did not affect IL-10 production in T cells. This discrepancy may be due to their distinct mechanisms of action—oligomycin blocks proton translocation and inhibits ATP synthesis directly, leading to energy deprivation, while Bz-423 binds to the oligomycin sensitivity-conferring protein subunit and induces mitochondrial reactive oxygen species (ROS), which may sustain IL-10 expression. DCA, which shifts cellular metabolism from glycolysis toward oxidative phosphorylation, most prominently increased IL-10-producing CD4⁺ T cells compared to the control group. In addition, etomoxir and metformin do not affect IL-10 production.

**Figure 2.**
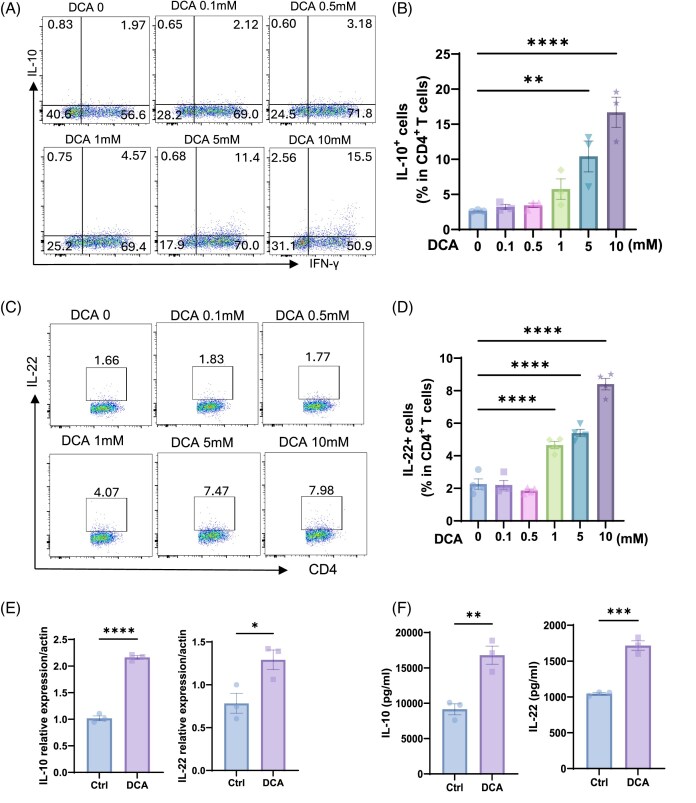
DCA promotes IL-10 and IL-22 production in a dose-dependent manner. (**A**–**D**) CD4⁺ T cells were stimulated under Th1-polarizing conditions with increasing concentrations of DCA (0–10 mM) for 5 days. (**A, B**) Intracellular staining was performed to assess IL-10^+^ CD4^+^ T cells (*n* = 3/group). (**C, D**) Intracellular staining was performed to assess IL-22^+^ CD4^+^ T cells (*n* = 4/group). (**E**) mRNA levels of *IL10* and *IL22* were measured by RT-qPCR at 24 h (*n* = 3/group). (**F**) Supernatants were collected at 72 h and analyzed for IL-10 and IL-22 by ELISA (*n* = 3/group). Representative of three experiments. Data are presented as mean ± SEM. One-way ANOVA with Bartlett’s multiple comparisons test (B and D); unpaired Student’s *t*-test (E and F). *P < 0.05, **P < 0.01, ***P < 0.001, ^****^P < 0.0001.

These results indicate that metabolic pathways differentially modulate T cell production of IL-10. To determine the dose-dependent effects of DCA on IL-10 production in Th1 cells, splenic CD4⁺ T cells were cultured under Th1-polarizing conditions in the presence of increasing concentrations of DCA (0–50 mM) for 5 days. We found that DCA increased IL-10 expression in a dose-dependent manner from 0 to 10 mM (Fig. 2A and B). However, DCA at 50 mM suppressed T cell viability (supplementary Fig. 1, see online supplementary material). Interestingly, we found that DCA also increased T cell production of IL-22 in a dose-dependent manner (Fig. 2C and D). Therefore, we chose 10 mM as the optimized dose for the subsequent experiments.

At the transcriptional level, quantitative PCR analysis confirmed that DCA significantly upregulated IL-10 and IL-22 mRNA expression compared to untreated Th1 cells after 24 h (Fig. 2E). Consistently, DCA increased secreted IL-10 and IL-22 protein levels in T cell culture supernatants (Fig. 2F).

These findings reveal that DCA modulates CD4⁺ T cell expression of IL-10 and IL-22, demonstrating a novel metabolic mechanism by which DCA reprograms Th1 cell function, with potential implications for therapeutic modulation of immune responses in inflammatory diseases.

We next explored whether DCA affects IFN-γ-producing Th1 and IL-17A-producing Th17 cell differentiation. To this end, we cultured CD4^+^ T cells with or without DCA under Th1 or Th17 conditions. We found that DCA increased IFN-γ production but did not affect IL-17A production in T cells under Th1 and Th17 conditions, respectively (supplementary Fig. 2A–C, see online supplementary material). Additionally, we found that DCA upregulated IL-22 but not IL-10 in T cells under Th17 conditions (supplementary Fig. 2D–F).

### DCA promotes T cell production of IL-22 and IL-10 via Ahr and Bhlhe40, respectively

**Figure 3.**
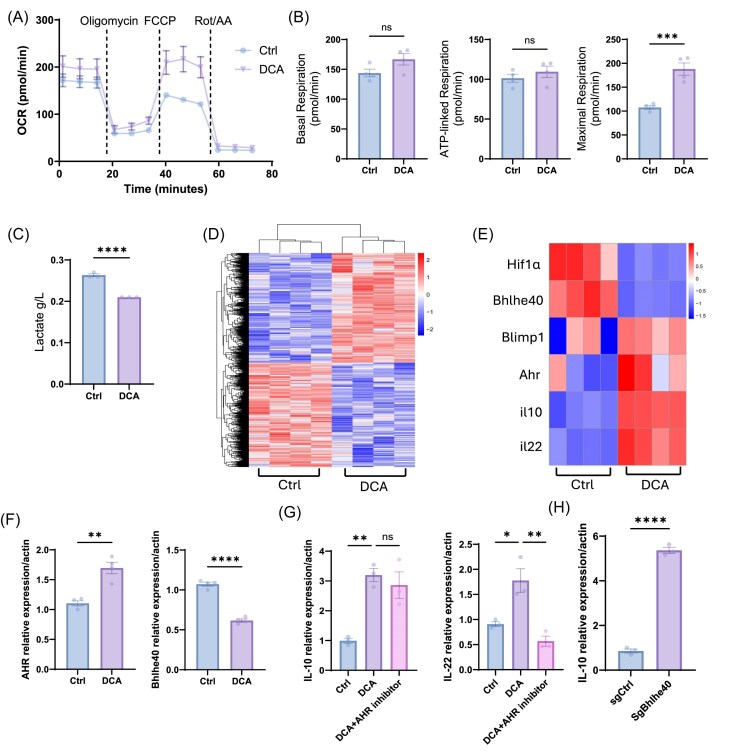
DCA regulates IL-10 and IL-22 expression through Bhlhe40 and Ahr. (**A, B**) Different oxygen consumption levels in DCA-treated Th1 cells and control Th1 cells (*n* = 4/group). (**C**) Lactate levels (*n* = 3/group) were determined in culture supernatants. (**D, E**) RNA-seq analysis was conducted on CD4⁺ T cells cultured under Th1 conditions ± DCA for 48 h (*n* = 4/group). (**D**) Heatmap visualization of overall transcriptomic changes. (**E**) Highlighted expression of *IL10, IL22, Ahr, Bhlhe40, Hif1a*, and *Blimp1*. (**F**) RT-qPCR was performed to measure *Ahr* and *Bhlhe40* mRNA levels (*n* = 4/group). (**G**) RT-qPCR analysis of *IL10* and *IL22* mRNA levels with or without DCA (10 mM)/Ahr inhibitor (CH-223191, 3 µM) at 24 h (*n* = 3/group). (**H**) CRISPR-mediated deletion of *Bhlhe40* was used to assess effects on *IL10* transcription (*n* = 3/group). Representative of three experiments. Data are presented as mean ± SEM. Unpaired Student’s *t*-test (B, C, and F); one-way ANOVA with Bartlett’s multiple comparisons test (G and H). ns, not significant; *P < 0.05, **P < 0.01, ***P < 0.001, ^****^P < 0.0001.

To confirm the effect of DCA on T cell metabolism, we analyzed mitochondrial function using a Mito Stress kit by Seahorse Analyzer. We found that DCA-treated Th1 cells demonstrated a higher level of maximal oxygen consumption but a similar level of basal and ATP-related respiration (Fig. 3A and B). Additionally, we measured the lactate levels in T cell culture supernatants using a Cedex Bio Analyzer, which showed that DCA suppressed Th1 cell secretion of lactate (Fig. 3C). Bulk RNA-seq was performed on CD4⁺ T cells cultured with or without DCA under Th1 conditions. Unsupervised hierarchical clustering revealed that DCA treatment dramatically regulates the Th1 cell transcriptional program with distinct gene expression profiles between control and DCA-treated cells (Fig. 3D). RNA-sequencing data confirmed that *IL10* and *IL22* were increased after treatment with DCA (Fig. 3E).

To investigate the transcriptional regulation by which DCA modulates IL-10 and IL-22 expression, we analyzed our bulk RNA-seq dataset for key known transcription factors, including Ahr, Blimp1 (*Prdm1*), Hif1a, and Bhlhe40. We found that DCA did not change the expression of *Prdm1* and suppressed *Hif1a* expression, suggesting that these genes did not mediate DCA upregulation of IL-10 and IL-22. In contrast, *Ahr* was upregulated and *Bhlhe40* was downregulated in response to DCA (Fig. 3E). Consistently, PCR confirmed that DCA increased Ahr expression and reduced Bhlhe40 expression in T cells (Fig. 3F).

Ahr has been shown to promote both IL-10 and IL-22 production in T cells [32]. First, we determined whether enhanced Ahr expression mediates DCA induction of T cell IL-10 and IL-22 production. We added an Ahr inhibitor, CH-223191, to T cell cultures in the presence or absence of DCA. The inhibition of Ahr abrogated DCA-induced IL-22 transcription but did not significantly affect IL-10 mRNA levels (Fig. 3G), indicating that enhanced Ahr mediates DCA promotion of T cell IL-22 production, whereas IL-10 is regulated through additional mechanisms.

Bhlhe40, a family of basic helix–loop–helix transcriptional regulators that bind E-box DNA motifs [33], has been shown to serve as a negative regulator for T cell IL-10 production in various settings [34]. To determine whether DCA inhibition of Bhlhe40 mediates DCA induction of T cell IL-10 production, we used CRISPR-Cas9 RNP to knock out Bhlhe40 in CD4⁺ T cells. Loss of Bhlhe40 significantly increased *IL10* compared to control T cells (Fig. 3E), suggesting that DCA downregulation of Bhlhe40 mediates DCA induction of T cell IL-10 production. These results demonstrate that DCA enhances IL-22 and IL-10 production by differentially modulating the Ahr and Bhlhe40 transcriptional factors.

### DCA-treated Th1 cells suppress naïve T cell proliferation

**Figure 4.**
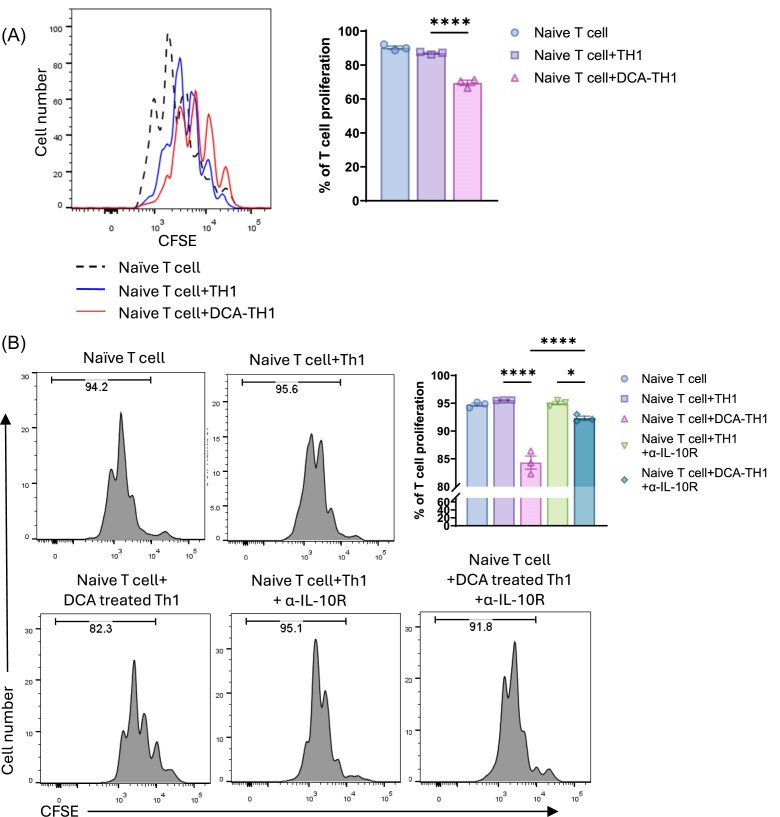
DCA-treated Th1 cells suppress naïve T cell proliferation via IL-10. (**A**) CFSE-labeled CD45.2⁺ naïve CBir1 CD4⁺ T cells were cultured alone or co-cultured with untreated or DCA-treated Th1 cells (ratio 1 : 0.5) for 48 h. T cell proliferation was assessed by CFSE dilution. Left: representative CFSE histograms. Right: quantification of T cell proliferation. (**B**) Naïve T cells were co-cultured in the presence or absence of 5 µg/ml anti–IL-10 receptor (α–IL-10R) antibody. T cell proliferation was assessed by CFSE dilution. Left: representative CFSE histograms. Right: quantification of T cell proliferation. Representative of three experiments. Data are presented as mean ± SEM. One-way ANOVA with Bartlett’s (A) or Tukey’s (B) multiple comparisons test. *P < 0.05, ^****^P < 0.0001.

It has been well-established that T cell production of IL-10 inhibits T cell function [35, 36]. To investigate the regulatory effects of DCA-treated T cells on naïve T cell proliferation, we pretreated CD45.2^+^ T cells with or without DCA under Th1 conditions for 5 days. These Th1 cells were then co-cultured with carboxyfluorescein succinimidyl ester (CFSE)-labeled CD45.1^+^ naïve CD4⁺ T cells. While control Th1 cells did not affect naïve T cell proliferation, DCA-treated Th1 cells significantly suppressed their proliferation (Fig. 4A). IL-10 has been shown to inhibit naïve T cell proliferation [37]. To determine whether this suppression was IL-10 dependent, an IL-10 receptor (IL-10R)-blocking antibody was applied. Blocking IL-10R restored the proliferative capacity of naïve T cells co-cultured with DCA-treated Th1 cells, indicating that IL-10 mediates the suppressive effect of DCA-treated Th1 cells on T cell proliferation (Fig. 4B). These data suggest that DCA-treated Th1 cells acquire immunosuppressive properties through IL-10 production, limiting naïve T cell responses.

### Treatment with DCA inhibits *C. rodentium*-induced intestinal inflammation

**Figure 5.**
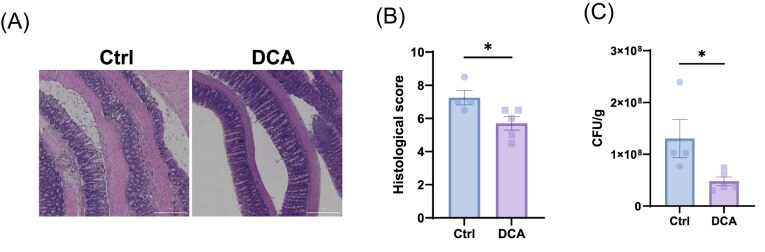
DCA treatment ameliorates *C. rodentium*-induced colitis. Wild-type mice were orally infected with *C. rodentium* (5 × 10⁸ CFU/mouse) on day 0. Mice received either water or DCA (5 mg/mouse) daily by oral gavage (control: *n* = 4, DCA: *n* = 5). (**A**) H&E staining of the colon for histological assessment. (**B**) Histopathology scores were determined in a blinded manner. (**C**) Fecal bacterial load was measured on day 7 post-infection. Representative of three experiments. Data are presented as mean ± SEM. Mann–Whitney U test (B); unpaired Student’s t-test (C). *P < 0.05.

T cell production of IL-10 and IL-22 is crucial in the maintenance of intestinal homeostasis and protection from intestinal inflammation [38–41]. As DCA promotes T cell production of IL-10 and IL-22, we then investigated whether treatment with DCA inhibits intestinal inflammation. The mice were infected with *C. rodentium* and treated with or without DCA in drinking water. *Citrobacter rodentium* infection induced severe colitis, as demonstrated by weight loss, intense infiltrates, severe inflammation, and histopathology (Fig. 5A and B). The mice receiving DCA exhibited significantly attenuated weight loss compared to untreated controls (Fig. 5A). Histological examination of colonic tissues showed reduced inflammation and epithelial damage in DCA-treated mice, as reflected by lower histological scores (Fig. 5B). DCA treatment significantly reduced fecal bacterial burden on day 7 post-infection (Fig. 5C). These findings demonstrate that DCA protects against intestinal inflammation, highlighting a novel role of metabolic modulation in mucosal immunity.

## Discussion

It has been well-established that different metabolic programs regulate the differentiation and function of different subsets of T cells. Effector T cells mainly depend on increased aerobic glycolysis for energy and biosynthetic precursors, while Treg cells depend predominantly on mitochondrial oxidation for energy and biosynthetic precursors [13–17]. However, the metabolic programs regulating individual cytokine production in T cells are still largely unclear. Our current study demonstrated that treatment with DCA, which decreases T cell metabolism with a greater effect on glycolysis than OXPHOS, promoted T cell production of IL-10 and IL-22, the crucial cytokines in protecting against intestinal inflammation.

Dysregulated T cell responses to gut microbiota mediate the pathogenesis of IBD. While T cells produce proinflammatory cytokines, which contribute to the induction of colitis, they also produce anti-inflammatory cytokines, including IL-10 and IL-22, which inhibit colitis development. Effector T cell IL-10 production has been considered a self-regulatory mechanism to restrain an excessive T cell response in the intestines, thereby preventing intestinal damage. Indeed, genetic polymorphisms of the IL-10 locus have been reported to be associated with increased susceptibility to IBD [42, 43]. Consistently, deficiency in IL-10 or IL-10R results in severe intestinal inflammation in both mice and humans [44–47]. IL-10–IL-10R signaling, in Treg cells, effector T cells, and innate immune cells, plays a central role in maintaining intestinal homeostasis [48–50]. T cell production of IL-22 contributes to wound healing in the intestines and induces epithelial cell production of antimicrobial peptides, including RegIIIγ and β-defensins [40, 51, 52]. Thus, identifying the metabolic pathways promoting IL-10 and IL-22 could provide novel targets for treating IBD by enhancing such pathways. As treatment with DCA promotes T cell 1L-10 and IL-22 production, which has been shown to decrease T cell metabolism with a greater effect on glycolysis than OXPHOS, it will be worth exploring the possibility of promoting T cell IL-10 and IL-22 production through enhancing mitochondrial OXPHOS. It has been shown that the higher status of mitochondrial OXPHOS in Tregs is crucial for their energy and biosynthetic precursors. Our studies thus also beg the question of whether a high mitochondrial OXPHOS status is required for T cells with regulatory function.

Different transcription factors control IL-10 and IL-22 production. Ahr has been shown to be crucial for IL-22 production in both T cells and ILC3. Our data indicated that DCA treatment induced T cell expression of Ahr, and inhibition of Ahr by an Ahr inhibitor suppressed DCA induction of IL-22 production, suggesting a crucial role of Ahr in mediating DCA induction of T cell IL-22 production. Hif1α has been shown to increase IL-22 expression and promote glycolysis in CD4⁺ T cells. Here, we found that DCA suppressed Hif1a, which is consistent with the known effect of DCA on inhibiting glycolysis. However, despite decreased Hif1a levels, IL-22 expression was increased after treatment with DCA, indicating that DCA suppressed glycolysis and Hif1a is unlikely to upregulate IL-22 expression. Some transcription factors have been implicated in driving T cell IL-10 production, such as *Prdm1* (encodes Blimp1) [53–56], while Bhlhe40 negatively regulates IL-10 production [34]. DCA did not affect the expression of *Prdm1*. However, it inhibited *Bhlhe40* expression. Knockdown of *Bhlhe40* promoted T cell IL-10 production, suggesting that DCA induction of T cell IL-10 production is mediated by inhibition of *Bhlhe40* but not through Blimp1.

DCA has been used in treating human diseases, including hereditary mitochondrial metabolic diseases and lactic acidosis, for a long time [26, 57, 58]. DCA has also been actively investigated for treating various cancers and autoimmune diseases [59–61]. Our studies demonstrate that DCA treatment inhibited colitis development in the *C. rodentium* infection model. It will be interesting to further investigate whether DCA can also inhibit colitis development in other experimental colitis models. While our findings support the potential of DCA as a therapeutic agent in IBD, several translational challenges need to be addressed. First, the dose and treatment duration required for efficacy in chronic intestinal inflammation remain to be defined. Second, the safety profile of long-term DCA administration, particularly regarding hepatotoxicity and neurotoxicity, warrants careful evaluation. Third, because DCA broadly influences cellular metabolism, off-target effects on other immune and non-immune cell populations may complicate its therapeutic application. Addressing these issues in preclinical and clinical studies will be essential for translating the protective effects of DCA into a treatment strategy for IBD. Importantly, dose–response studies *in vivo* and assessment of toxicity of DCA would improve translational relevance. In addition to its protective role in colitis, our findings suggest that DCA also enhance host defense against *C. rodentium* infection. However, the specific mechanism by which it does so remains unclear, due to the lack of detections related to the mucosal barrier, inflammatory factors, and other relevant parameters. Future studies will be required to determine whether DCA can broadly augment resistance to other intestinal infections and whether its immunomodulatory effects can be harnessed therapeutically to prevent pathogen-driven exacerbation of intestinal inflammation.

In summary, our studies demonstrate that DCA promotes a metabolic program that regulates T cell production of IL-10 and IL-22, and that DCA treatment inhibits colitis development. However, it remains unclear whether the observed protection is solely mediated by IL-10 and IL-22 derived from DCA-treated T cells. Further investigation will be required to directly determine the contribution of DCA-modulated T cell cytokine production to protection against *C. rodentium*–induced intestinal inflammation.

## Supplementary Material

pbaf025_Supplemental_File
