# Supplementary material for: Metabolic regulation of T cell production of IL-10 and IL-22 protects against intestinal inflammation
Source: Precis Clin Med. 2025 Oct 24;8(4):pbaf025. doi: 10.1093/pcmedi/pbaf025 (PMC12658364; doi:10.1093/pcmedi/pbaf025)

**Supplementary Data**

**
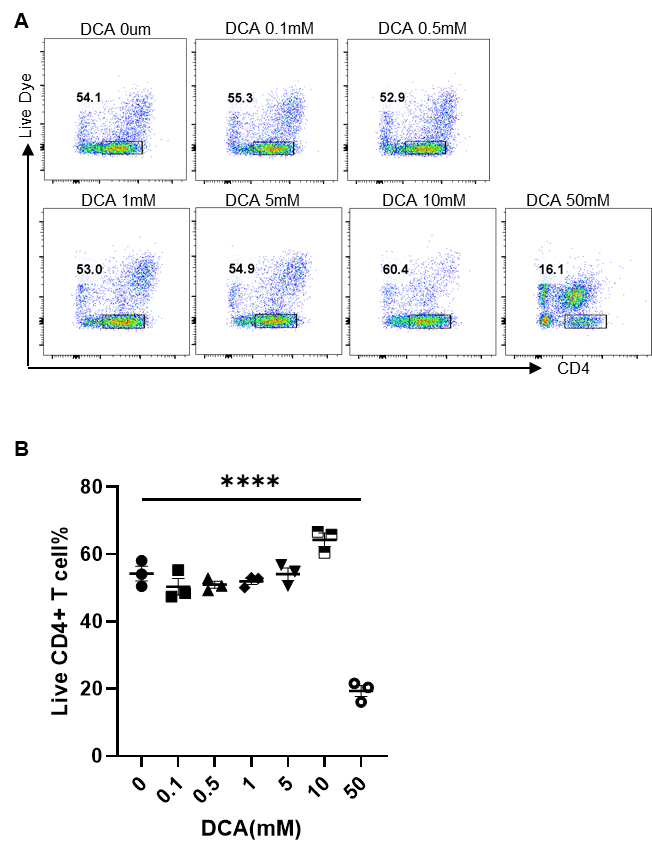
**

**Supplementary Figure 1. The effect of different doses of DCA on T cell viability.** CD4^+^ T cells were activated and treated with DCA at the doses indicated. **(A)** Representative flow cytometry. **(B)** Statistical quantification of Live CD4^+^ T cells. Representative of three experiments. Data are presented as mean ± SEM. One-way ANOVA with Bartlett's multiple comparisons test (B). *****P* < 0.0001.


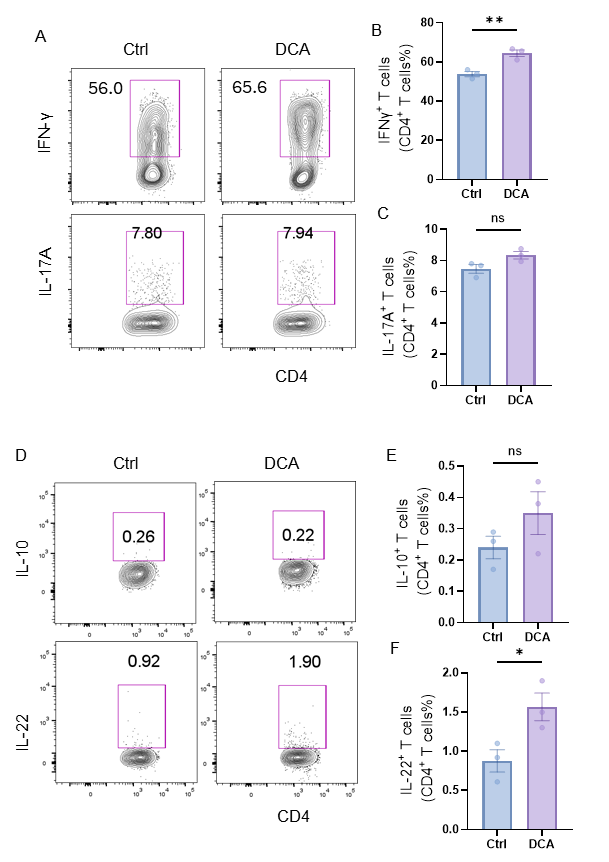


**Supplementary Figure 2. The effect of DCA on T cells. (A-B)** CD4^+^ T cells were activated and treated with DCA (10 mM) under Th1 or Th17 conditions for 5 days. Representative flow cytometry **(A)**. Statistical quantification of IFN-γ^+^ **(B)** and IL17A^+^ **(C)** CD4^+^ T cells. **(D-F)** CD4^+^ T cells were activated and treated with DCA (10 mM) under Th17 conditions for 5 days. Representative flow cytometry **(A)**. Statistical quantification of IL-10^+^ **(E)** and IL-22^+^ **(F)** CD4^+^ T cells. Statistical quantification of IL-17A^+^ CD4^+^ T cells. Representative of three experiments. Data are presented as mean ± SEM. Unpaired Student’s t-test. **(B)**. Ns, not significant; ***P* < 0.01.

**Supplementary Table 1. Primers for qRT-PCR.**


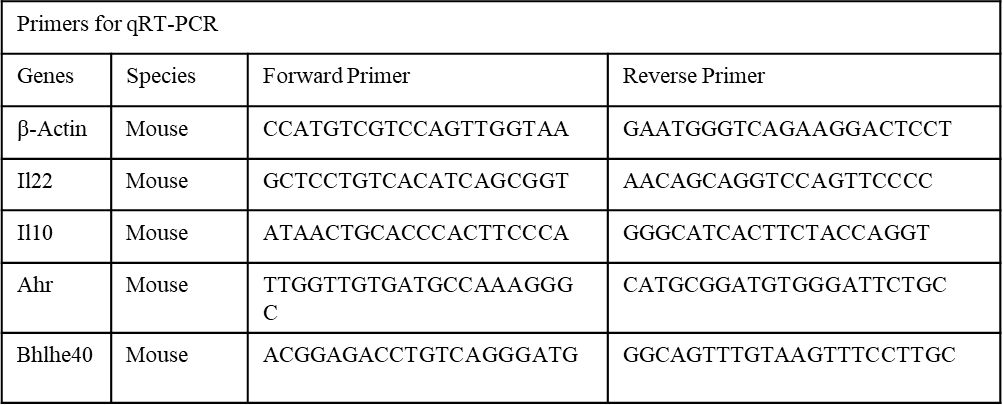

Supplement: pbaf025_Supplemental_File [file pbaf025_supplemental_file.docx]
